# Structural basis of GABA_B_ receptor–G_i_ protein coupling

**DOI:** 10.1038/s41586-021-03507-1

**Published:** 2021-04-28

**Authors:** Cangsong Shen, Chunyou Mao, Chanjuan Xu, Nan Jin, Huibing Zhang, Dan-Dan Shen, Qingya Shen, Xiaomei Wang, Tingjun Hou, Zhong Chen, Philippe Rondard, Jean-Philippe Pin, Yan Zhang, Jianfeng Liu

**Affiliations:** 1 ZJU-HUST Joint Laboratory of Cellular Signaling, Key Laboratory of Molecular Biophysics of MOE, International Research Center for Sensory Biology and Technology of MOST, College of Life Science and Technology, Huazhong University of Science and Technology (HUST), Wuhan, China; 2 Department of Biophysics and Department of Pathology of Sir Run Run Shaw Hospital, Zhejiang University School of Medicine, Hangzhou, China; 3 Liangzhu Laboratory, Zhejiang University Medical Center, Hangzhou, China; 4 Zhejiang Provincial Key Laboratory of Immunity and Inflammatory Diseases, Hangzhou, China; 5 Bioland Laboratory, Guangzhou Regenerative Medicine and Health Guangdong Laboratory, Guangzhou, China; 6 Innovation Institute for Artificial Intelligence in Medicine of Zhejiang University, College of Pharmaceutical Sciences, Zhejiang University, Hangzhou, China; 7 Key Laboratory of Neuropharmacology and Translational Medicine of Zhejiang Province, Zhejiang Chinese Medical University, Hangzhou, China; 8 Institut de Génomique Fonctionnelle (IGF), Université de Montpellier, CNRS, INSERM, 34094 Montpellier, France; 9 MOE Frontier Science Center for Brain Research and Brain-Machine Integration, Zhejiang University School of Medicine, Hangzhou, China

**Keywords:** Membrane structure and assembly, G protein-coupled receptors, Cryoelectron microscopy

## Abstract

G-protein-coupled receptors (GPCRs) have central roles in intercellular communication^1,2^. Structural studies have revealed how GPCRs can activate G proteins. However, whether this mechanism is conserved among all classes of GPCR remains unknown. Here we report the structure of the class-C heterodimeric GABA_B_ receptor, which is activated by the inhibitory transmitter GABA, in its active form complexed with G_i1_ protein. We found that a single G protein interacts with the GB2 subunit of the GABA_B_ receptor at a site that mainly involves intracellular loop 2 on the side of the transmembrane domain. This is in contrast to the G protein binding in a central cavity, as has been observed with other classes of GPCR. This binding mode results from the active form of the transmembrane domain of this GABA_B_ receptor being different from that of other GPCRs, as it shows no outside movement of transmembrane helix 6. Our work also provides details of the inter- and intra-subunit changes that link agonist binding to G-protein activation in this heterodimeric complex.

## Abstract (web summary)

Cryo-electron microscopy structure of heterodimeric GABA_B_ receptor in complex with G_i1_ protein reveals that the mode of G-protein binding in this class-C G-protein-coupled receptor differs from that of other classes.

## Main

GPCRs are essential elements that are involved in cell–cell communication and represent major targets for therapeutic drugs^1^. Recent structural studies have provided important information on how GPCRs can act as nucleotide-exchange factors that allow the release of GDP from the inactive G protein, and then the activation of these proteins upon GTP binding^2^. Several previous structures of activated GPCR–G protein complexes have revealed a similar mode of action for each^3–6^. Despite differences in the interaction mode of G proteins for various class-A, -B and -F GPCRs, in all previously characterized interactions the C-terminal extremity of the Gα subunit engages with a cavity on the intracellular side of the receptor that results from the opening of this domain owing to the movement of transmembrane helix (TM) 6 relative to TM3^5,7^.

Compared to other classes of GPCRs that can be activated in a monomeric form, class-C GPCRs are mandatory dimers^8^ that are composed of two identical or similar subunits^9–11^. These dimers may activate only one G protein at a time^10,11^, but the molecular basis of this asymmetric mode of action remains unknown. Among the class-C GPCRs that are activated by the neurotransmitter GABA, the GABA_B_ receptor (hereafter referred to as GABA_B_) is an attractive drug target for the treatment of brain diseases^12^. GABA_B_ is composed of two distinct subunits: GB1 (to which agonists bind) and GB2 (which is responsible for G-protein activation)^9,13,14^. Each subunit is composed of an extracellular Venus flytrap (VFT) domain and a transmembrane domain (TMD)^10,15^. The structure of this receptor has recently been solved in a number of states, including apo, antagonist-bound, agonist-bound, and agonist- and positive allosteric modulator (PAM)-bound^11,16–18^. Although these studies have helped to identify the conformational changes in subunits that are associated with ligand binding, it remains unclear at the atomic level how this heterodimeric GPCR activates G proteins.

Here we report the cryo-electron microscopy (cryo-EM) structure of the agonist- and PAM-bound form of the GABA_B_ in complex with the G protein G_i1_ at 3.5 Å resolution. Our results reveal a mode of G-protein coupling that differs from those that have previously been reported for GPCRs of other classes; our structures reveal that small movements of TM3 and TM5 lead to changes in the intracellular loops (ICLs) that offer a binding site for the G protein on the side of the GB2 subunit of GABA_B_. These data also help to refine models that describe how agonist binding in the VFT domain of GB1 leads to the activation of the TMD of GB2, and how small molecules can act as PAMs.

## Overall architecture of GABA_B_–G_i_ complex

**Fig. 1:**
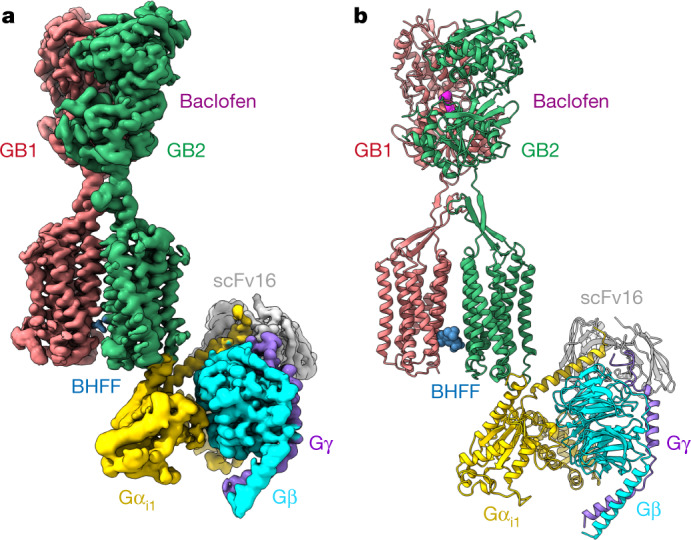
Cryo-EM structure of GABA_B_–G_i_ complex. **a**, **b**, Cryo-EM map (**a**) and model (**b**) of the baclofen- and BHFF-bound GABA_B_–G_i1_ complex.

**Extended Data Fig. 1:**
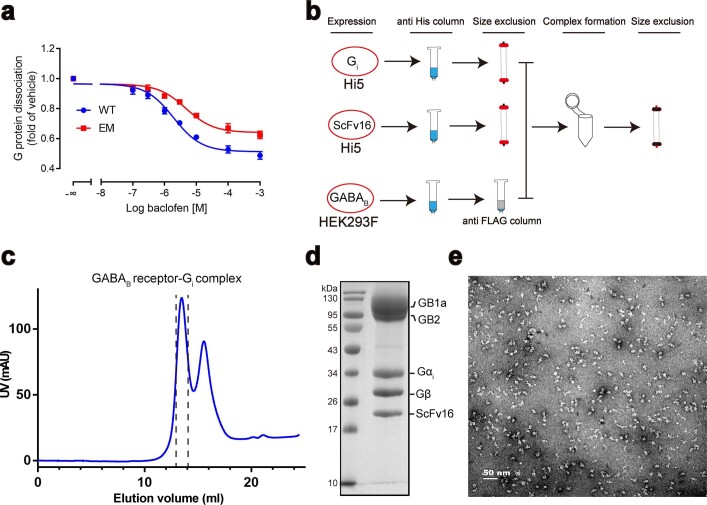
Purification of the GABA_B_–G_i1_ complex. **a** Pharmacology of wild-type GABA_B_ and the purification construct (EM) in a baclofen-mediated NanoBiT-G-protein dissociation assay. Data are mean ± s.e.m. from four independent experiments, performed in technical triplicate. **b**, Flow chart of the purification steps for the GABA_B_–G_i1_ complex. GABA_B_ was expressed in HEK293F cells. Heterotrimeric G_i1_ and scFv16 were expressed in Hi5 cells. **c**–**e**, Size-exclusion chromatography profile (**c**), SDS–PAGE gel (**d**) and the negative-staining electron microscopy analysis (**e**) of the purified GABA_B_–G_i1_ complex. Source data

**Extended Data Fig. 2:**
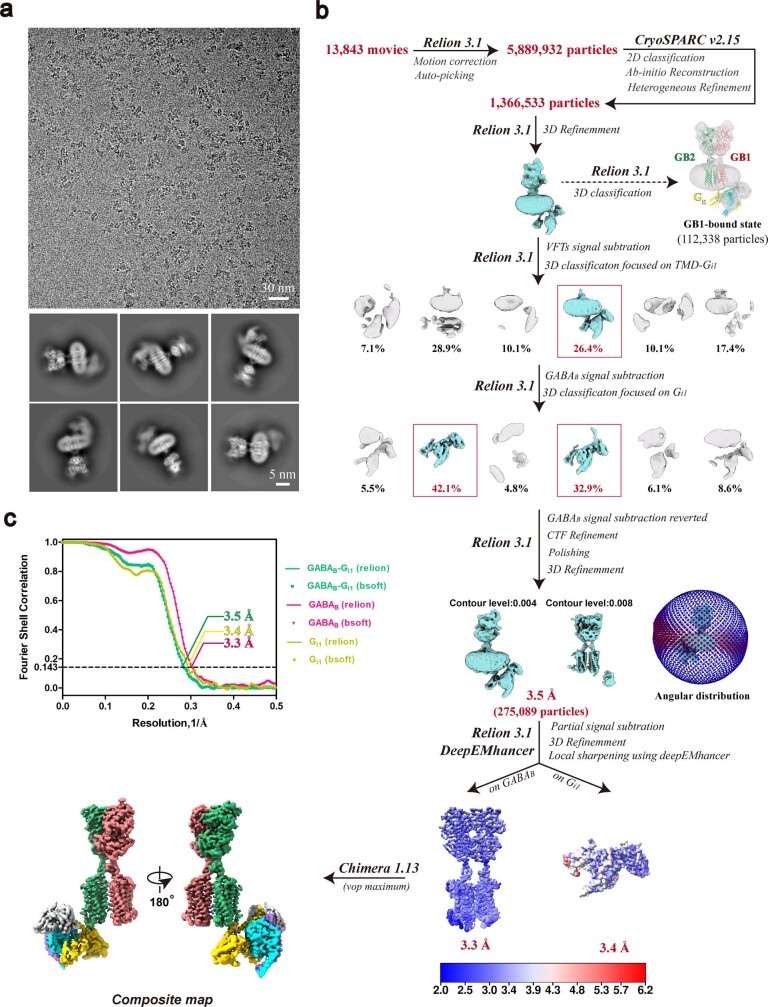
Cryo-EM data processing of the GABA_B_–G_i_ complex. **a**, Representative cryo-EM micrograph (from 13,483 movies) and 2D class averages (from 16 classes) of the GABA_B_–G_i1_ complex. **b**, Flow chart of cryo-EM data processing. **c**, Gold-standard Fourier shell correlation curves of the globally refined GABA_B_–G_i1_ complex and the locally refined GABA_B_ and G_i1_.

**Extended Data Fig. 3:**
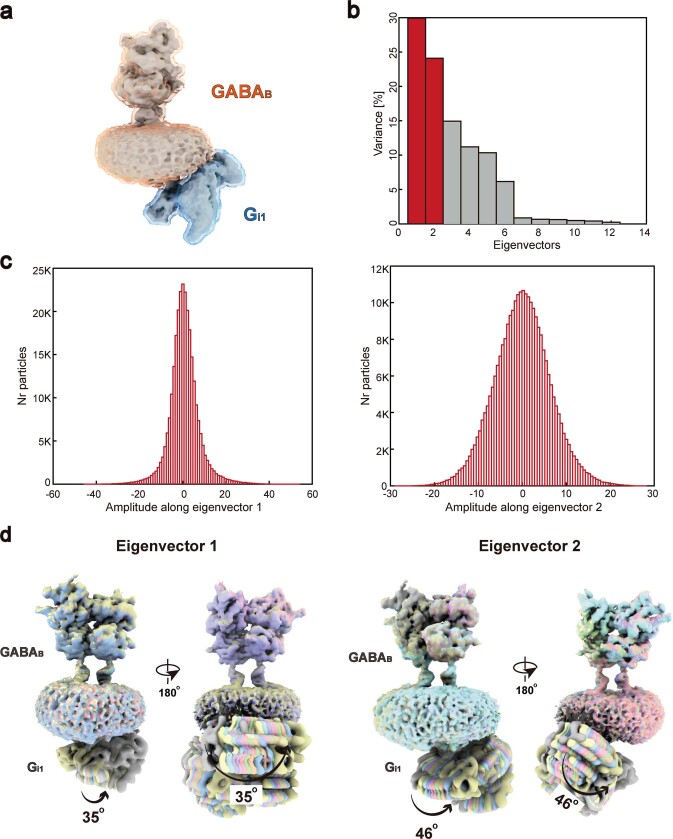
Flexibility analysis of GABA_B_–G_i1_ coupling. **a**, Multibody refinement and principal component analysis of the relative orientations of GABA_B_ and G_i1_. The GABA_B_–G_i1_ consensus map and the body masks of GABA_B_ and G_i1_ are shown. **b**, Contribution of individual eigenvectors to the total variance in rotation and translation between GABA_B_ and G_i1_. The first and second eigenvectors explain more than 50% of the variance observed and are highlighted in red. **c**, Histograms of the amplitudes along the first and second eigenvectors. **d**, Motion represented by the first and second eigenvectors.

**Extended Data Fig. 4:**
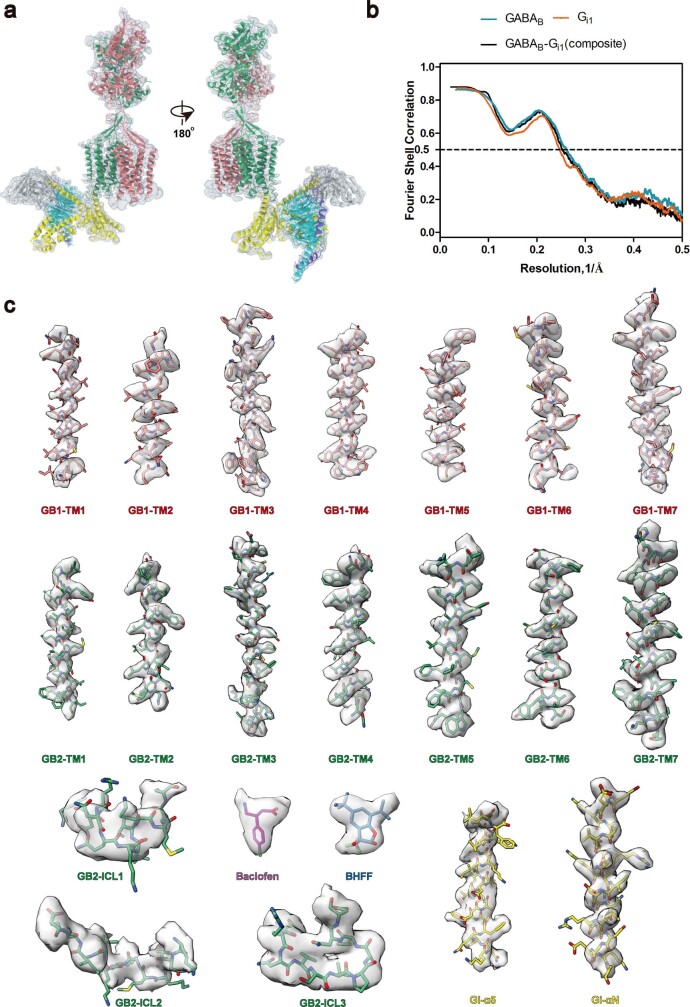
Analysis of the quality of the cryo-EM map. **a**, Global fitting of the GABA_B_–G_il_ structure into the composite cryo-EM density map. **b**, Fourier shell correlation curves of the model versus the map. **c**, Cryo-EM densities and the fitted atomic models are shown. GB1 in red; GB2 in green; Gα_i1_ in yellow; baclofen in magenta; BHFF in blue.

Using a modified version of a previously established protocol^11^ (Extended Data Fig. 1), we assembled the GABA_B_–G_il_ complex by incubating purified GABA_B_ with G_i1_ in the presence of the agonist baclofen and the PAM *R*,*S*-5,7-di-tert-butyl-3-hydroxy-3-trifluoromethyl-3*H*-benzofuran-2-one (BHFF)^19^ (Fig. 1a, b). Our cryo-EM analysis indicated that the binding of G_il_ to GABA_B_ was flexible, and the consensus refinement map exhibited poor density in the G protein (Extended Data Fig. 2). The flexible conformations of G_i1_ bound to GABA_B_ in a similar pocket, but were rotated within an angle of up to 46° (Extended Data Fig. 3, Supplementary Videos 1, 2). To obtain detailed structural information, we subjected the individual structures of G_il_ and GABA_B_ to local reconstruction, and produced improved G_il_ and the GABA_B_ maps at a resolution of 3.4 Å and 3.3 Å, respectively (Extended Data Figs. 2, 4). These maps were combined on the basis of the consensus refinement map, and provided a rational structural framework for analyses of G-protein coupling (Fig. 1a, b, Extended Data Table 1).

**Extended Data Fig. 5:**
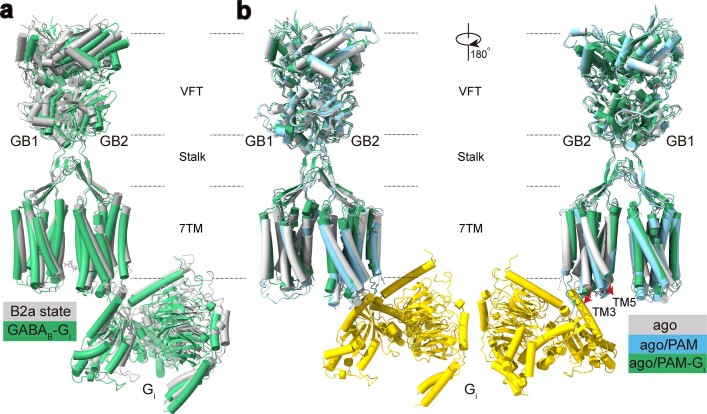
Structural comparisons of the determined GABA_B_–G_i_ complex with the previously reported low-resolution B2a state GABA_B_–G_i_ complex, the agonist-bound, and the agonist- and PAM-bound GABA_B_. **a**, Structural comparison between the low-resolution GABA_B_–G_i_ complex in B2a state (grey) and this study determined GABA_B_-G_i_ structure (green). **b**, Structural comparisons of the G_i_-bound GABA_B_ (ago/PAM–G_i_) (green) with the agonist-bound (ago) (PDB 6UO9) (grey) and agonist- and PAM-bound GABA_B_ (ago/PAM) (PDB 6UO8) (blue).

Our determined structure of the GABA_B_–G_il_ complex assumes an overall architecture that is similar to the previously reported low-resolution GABA_B_–G_il_ structure in the B2a state^11^ (Extended Data Fig. 5a). The agonist- and PAM–G_il_-bound GABA_B_ exhibited a conformation similar to that of the agonist- and PAM-bound GABA_B_ (Protein Data Bank code (PDB) 6UO8) with a root mean squared deviation of 2.3 Å, in which the TMDs adopted a TM6–TM6 interface and the TMD of subunit GB2 showed an outward shift at the intracellular ends of TM3 and TM5. We did not observe conformation changes of TM3 and TM5 in the agonist-bound states (PDB 6UO9) (Extended Data Fig. 5b). Our structure shows that G_il_ binds to a shallow cavity that is formed by the ICLs of GB2, which provides a structural basis for understanding the distinct mode of G_il_ coupling to GABA_B_.

## Asymmetric activation of GABA_B_

**Fig. 2:**
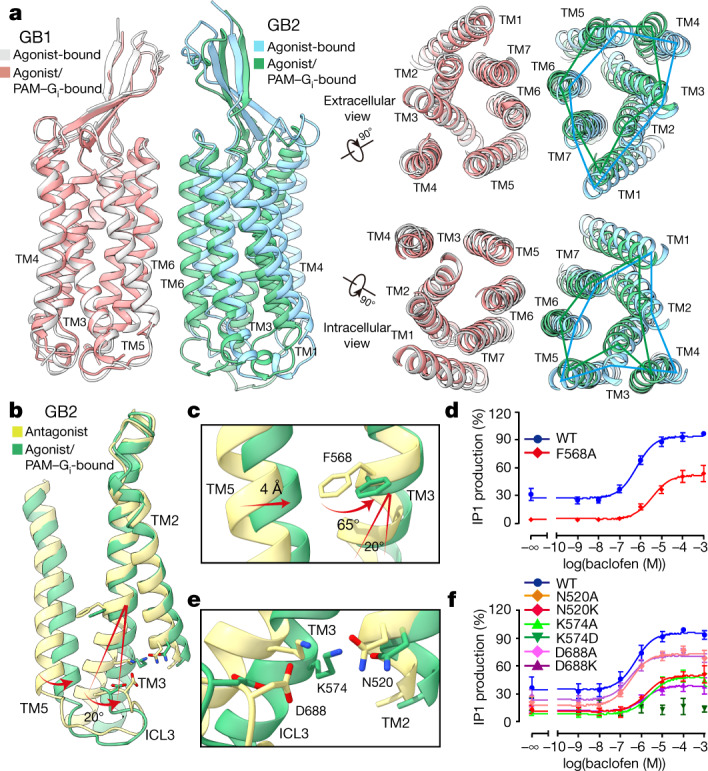
Asymmetric activation of GABA_B_. **a**, Side, extracellular, and intracellular views of the superposed structures of the agonist-bound (PDB 6UO9) and the agonist- and PAM-G_i_-bound (agonist/PAM-bound) GABA_B_, aligned by the TMD of GB1. **b**, Conformational changes of the TMD of GB2 between antagonist-bound (PDB 7C7S) and agonist- and PAM–G_i_-bound structure. **c**, Magnified views of the critical residue F568, the bulky side chain of which undergoes a substantial rotation upon activation and causes the TM3 shifting. **d**, Baclofen-induced IP1 accumulation of wild-type (WT) and F568A-mutant GABA_B_ using the chimeric Gα protein Gα_qi9_. Data are mean ± s.e.m. from six independent experiments, performed in technical triplicate. **e**, Magnified views of the ‘ionic lock’ located in the cytoplasmic TMD of GB2. **f**, Baclofen-induced IP1 accumulation of wild-type GABA_B_ and several forms of GABA_B_ with substitutions in the ionic lock region using Gα_qi9_. Data are mean ± s.e.m. from at least three independent experiments, performed in technical triplicate. Source data

**Extended Data Fig. 6:**
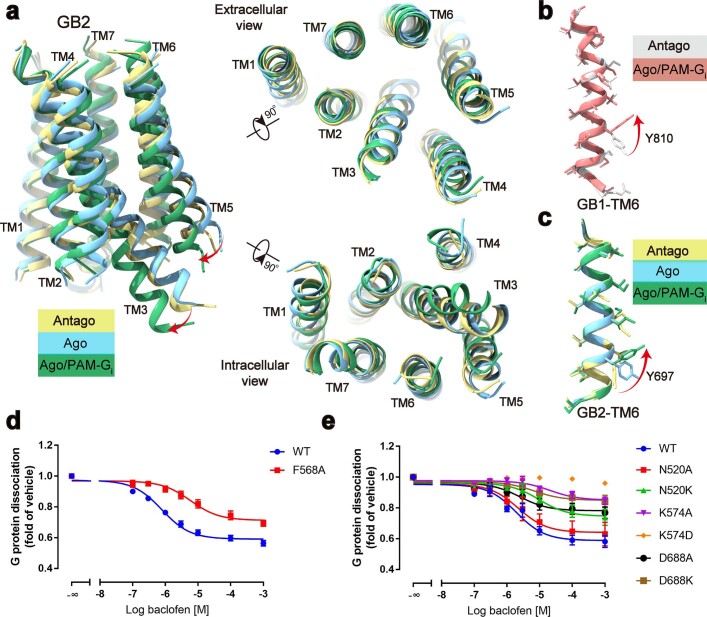
Intra-subunit conformational changes of the TMD of GB2 upon activation. **a**, Overlay of the structures of the TMD of GB2 in antagonist-bound (antago) (PDB 7C7S) (yellow), agonist-bound (ago) (PDB 6UO9) (sky blue), and agonist- and PAM–G_i_-bound (ago/PAM-G_i_) (green) states. **b**, **c**, Overlay of the different states of the TM6 of GB1 (antago, grey; ago/PAM–G_i_, red) (**b**) and the TM6 of GB2 (antago, yellow; ago, blue; ago/PAM–G_i_, green) (**c**). **d**, **e**, NanoBiT G-protein dissociation assay of GABA_B_ with alterations of the residues that are involved in activation. Data are mean ± s.e.m. from at least three independent experiments, performed in technical triplicate. Source data

In the GABA_B_–G_i1_ complex, there is no obvious opening of a central cavity on the intracellular side of the TMDs of either GB1 or GB2 (Fig. 2a, Extended Data Fig. 6a). Using the TMD of GB1 in agonist-bound GABA_B_ (PDB 6UO9) as a reference, the TMDs of GB1 remained unchanged but the TMDs of GB2 did not overlap well (a root mean squared deviation of 4.8 Å) (Fig. 2a). However, we observed only local environmental differences between two forms when the GB2 TMD alone was aligned (Extended Data Fig. 6a). The GB2 TMD underwent an anticlockwise rotation relative to GB1 upon binding to a PAM and/or G protein (Fig. 2a). Therefore, interactions with the PAM and G protein may induce further structural rearrangements to agonist-bound GABA_B_. GB1 Y810^6.44^ (superscript numbers refer to the GPCRdb numbering scheme) and GB2 Y697^6.44^ had rotamer changes and formed a hydrogen bond with GB2 N689^6.45^ and GB1 N811^6.45^, respectively (Extended Data Fig. 6b, c). The resulting TM6–TM6 interaction was critical for G-protein coupling but did not lead to a conformational change of TM6 relative to the rest of the GB2 TMD.

Intra-subunit conformational changes within the TMDs were located in the intracellular half of TM3 and the entire TM5 of GB2 (Fig. 2b). TM5 moved 4 Å towards TM3, and F568^3.44^ rotated away from TM5 by about 65° to avoid potential spatial clashes (which is likely to be a critical origin for the 20°- rotation of the cytoplasmic end of TM3) (Fig. 2c). Mutation of F568^3.44^ to alanine largely impaired GABA_B_-induced G_i_ coupling (Fig. 2d, Extended Data Fig. 6d), which suggests that the bulky side chain of F568^3.44^ is essential for GB2 activation. The intracellular tip of TM3 was farther away from TM5, and was further stabilized by three critical charged residues that may help to accommodate G protein (Fig. 2b, e). This is consistent with a previous study^20^ that identified residues of TM3 (K572^3.50^ and R575^3.53^) and TM6 (D688^6.35^) of GB2, all of which are conserved among class-C GPCRs. Similar to class-A GPCRs (in which a D/ERY motif constitutes an ionic lock that stabilizes the inactive state)^21^, K574^3.50^ and D688^6.35^of GB2 form an ionic lock in the inactive state and become weaker owing to inward movement of TM3 upon receptor activation. K574^3.50^ turned to N520^2.39^ of GB2 to form an additional ionic interaction (Fig. 2e). Substitution of these residues with alanine or oppositely charged amino acids impaired or abolished agonist-induced receptor activity (Fig. 2f, Extended Data Fig. 6e). The intra-subunit conformational changes of GB2 TMD led to asymmetric activation of GABA_B_ through binding and activation of a single G protein.

## Specificity of GABA_B_–G_i_ coupling

**Fig. 3:**
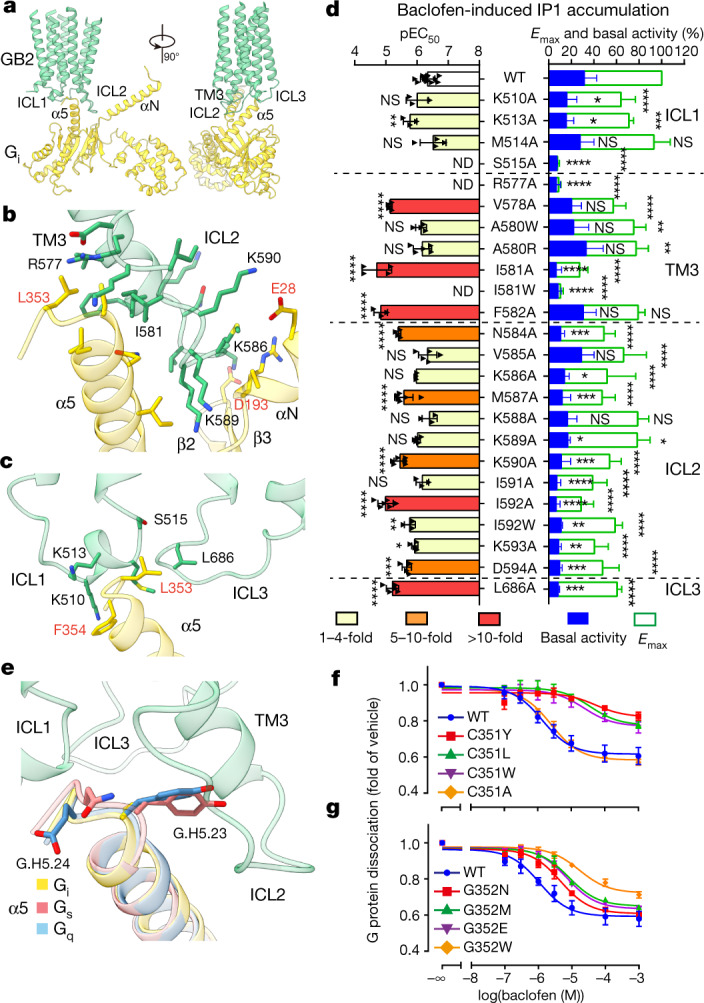
GABA_B_–G_i_ coupling and G-protein selectivity. **a**, The G_i1_ binding pocket in GABA_B_, which is mainly formed by three intracellular loops of GB2. GB2, green; Gα_i1_, yellow. **b**, **c**, Detailed interactions of the ICL2 and TM3 of GB2 with Gα_i_ (**b**), and of ICL1 and ICL3 with Gα_i_ (**c**). **d**, Baclofen-induced IP1 accumulation using Gα_qi9_. Bars represent differences in calculated *E*_max_ and basal activity or potency (pEC_50_) for each mutant as a percentage of the maximum in wild type. Data are mean ± s.e.m. from at least three independent experiments, performed in technical triplicate and analysed using one-way analysis of variance with Dunnett’s multiple comparison test to determine significance (compared with wild type). ND, not determined; NS, not significant. **e**, The C^G.H5.23^ and G^G.H5.24^ residues in the C-terminal α5 helix of Gα_i_ are involved in the selective coupling between GABA_B_ and G_i_ protein. The α5-helix structures of G_s_ (PDB 5VAI), G_q_ (PDB 6WHA) and the GABA_B_-bound G_i_ were aligned. **f**, **g**, Effect of C^G.H5.23^ (**f**) and G^G.H5.24^ (**g**) mutations in Gα_i_ on GABA_B_–G_i_ coupling using NanoBiT G-protein dissociation assay. Data are mean ± s.e.m. from at least three independent experiments. Source data

**Extended Data Fig. 7:**
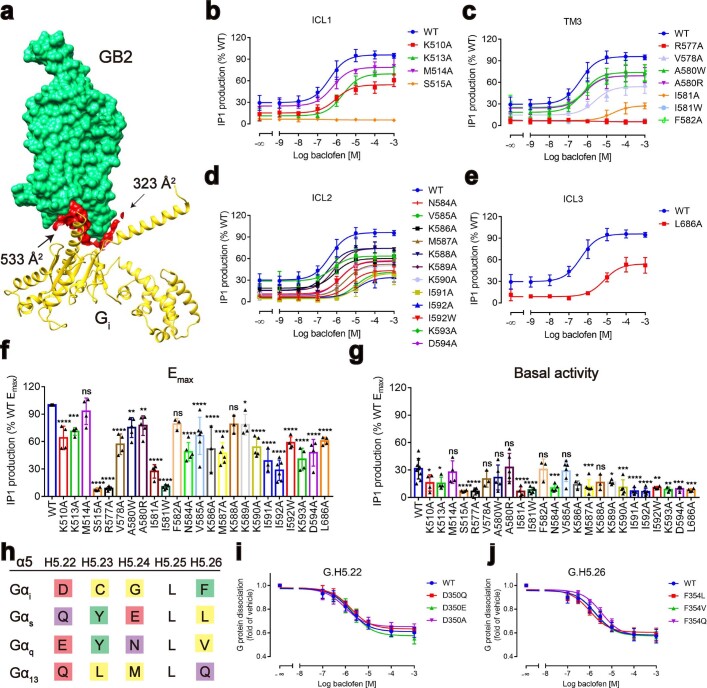
G_i_ activation and signalling assays. **a**, Interface of GABA_B_ and G_i1_ protein. GB2, green; G_i1_, yellow. The interaction interface between GB2 and G_i1_ is in red. **b**–**e**, Agonist-induced IP1 accumulation assay of the wild-type and the G_i1_-binding-pocket mutant GABA_B_. **f**, **g**, *E*_max_ and basal activity for each mutant relative to wild type, detected by IP1 accumulation assay and presented as dot plots. Data are mean ± s.e.m. from at least three independent experiments, performed in technical triplicate and analysed using one-way ANOVA with Dunnett’s multiple comparison test to determine significance (compared with wild type). **h**, Sequence alignment of the final five residues in the α5 helix among different Gα proteins. **i**, **j**, NanoBiT G-protein dissociation assay of the D350^G.H5.22^ (**i**) and F354^G.H5.26^ (**j**) mutant G_il_. Data points in **b**–**g**, **i**, **j** are mean ± s.e.m. from at least three independent experiments, performed in technical triplicate. Source data

In the GB2 subunit, the three ICLs and the intracellular tip of TM3 form a shallow pocket for the G protein (Fig. 3a, Extended Data Fig. 7a). The ICL2 of GB2 establishes extensive interactions with the α_5_ helix and the two linker regions (β_2_–β_3_ and α_N_–β_1_) in Gα_i1_ (Fig. 3b). There are potential salt bridges between lysine residues in this ICL2 (K586, K589 and K590) and acidic residues in α_N_ (E28) and the linker region in β_2_–β_3_ (D193) in Gα_i1_. ICL1 and ICL3 were away from the G protein and participated only in the recognition of the C-terminal ‘hook-like’ region of Gα_i_ (Fig. 3c).

Given the flexibility of G_i1_ engagement to the receptor, we subjected residues of GABA_B_ within 6 Å of the GABA_B_–G_i1_ interface to mutagenesis and functional analyses (Fig. 3d, Extended Data Fig. 7b–g, Supplementary Table 1). Substitutions of residues with alanine in the intracellular tip of TM3 and entire ICL2 of GB2 led to a substantial 20–75% reduction in maximal responses (*E*_max_) (Fig. 3d). Most mutants in the ICL2 showed decreased basal activity compared with wild type. Among them, M587A, K590A and I592A decreased the agonist potency (half-maximal effective concentration (EC_50_)) by 6–22 fold, which highlights the essential role of ICL2 in GB2–G_i1_ coupling (consistent with previous studies^22^). Substitutions in ICL1 (S515A) or TM3 (R577A and I581W) abolished GABA_B_-induced production of inositol monophosphate (IP1). Substitutions in TM3 (V578A, I581A, F582A and N584A) and in ICL3 (L686A) decreased the agonist potency by 9–32 fold, which indicates that ICL1, ICL3 and TM3 are involved in the recognition of G_i1_.

GABA_B_ predominantly couples to G_i/o_ subtypes of G protein^23^. The C-terminal 5–9 residues of the α_5_ helix of G protein have previously been found to be the key determinants for G-protein-coupling specificity^24,25^. The α_5_ helix of Gα_i1_ contributed 62% (533 Å^2^) of the interaction surface with GABA_B_ (Extended Data Fig. 7a). Sequence alignment of Gα_s_, Gα_q_, Gα_13_ and Gα_i_ showed four nonidentical amino acids among the final five C-terminal residues (G.H5.22–G.H5.26)^26^ (Fig. 3e, Extended Data Fig. 7h). We mutated these four residues in Gα_i1_ to the corresponding residues of Gα_s_, Gα_q_ and Gα_13_. Substitution mutations of C351^G.H5.23^ (superscript codes refer to common Gα numbering system) or G352^G.H5.24^—but not L353^G.H5.22^ or F354^G.H5.25^—impaired GABA_B_-induced G-protein signalling, which suggests an essential role for C351^G.H5.23^ and a partial involvement of G352^G.H5.24^ in the specificity of G_i_ coupling (Fig. 3f, g, Extended Data Fig. 7i, j), consistent with previous data^25^. The overall structure was similar in the backbone of the α_5_ helix to that in different G proteins, but Gα_s_ and Gα_q_ possess a tyrosine instead of cysteine in Gα_i_ G.H5.23, which may lead to potential steric hindrance with the ICL2 of GB2 (Fig. 3e). When replacing C351^G.H5.23^ or G352^G.H5.24^ with a bulky tryptophan to create clashes with ICL2, we observed decreased GABA_B_-induced G_i_ signalling, whereas the substitution of C351^G.H5.23^ with alanine led to no obvious signalling loss (Fig. 3f, g). The specificity of recognition of the α_5_ helix of G_i1_ by GABA_B_ confirmed the importance of ICL2 in the selective activation of G_i_.

## Distinct G_i_ binding model of GABA_B_

**Fig. 4:**
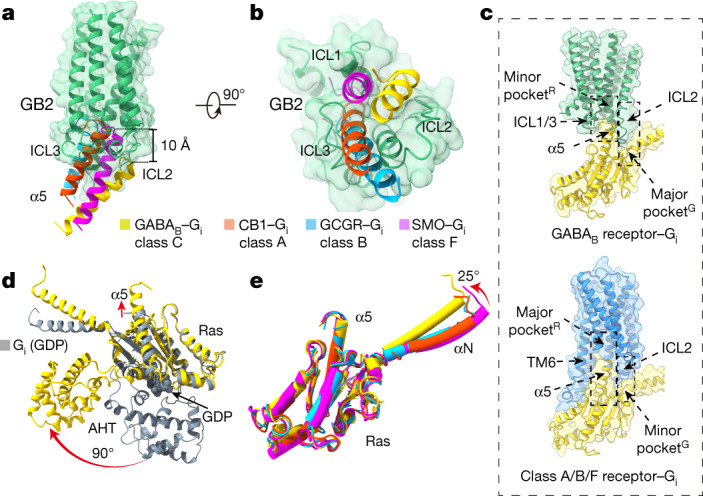
Distinct G_i_ binding model of GABA_B_. **a**, **b**, Orientations of the α5 helix in G_i_ protein when coupling to GABA_B_, cannabinoid receptor 1 (CB1) (class A), glucagon receptor (GCGR) (class B) and  smoothened (SMO) (class F). Structures were aligned by the TMDs; only the TMD of GB2 is shown, for clarity. GABA_B_-bound, yellow; CB1-bound α5, PDB 6N4B; GCGR-bound α5, PDB 6LML; SMO-bound α5, PDB 6OT0. **c**, Schematics of the two types of pocket that are involved in G-protein recognition. GABA_B_, green; monomeric GPCR, blue; G_i_, yellow. **d**, Superposition of GABA_B_-bound G_il_ with the GDP-bound G_il_. GDP-bound G_il_, PDB 1GP2. **e**, Structural comparison of the GABA_B_-bound G_il_ with CB1-, GCGR- and SMO-bound G_il_. G proteins are coloured as in **a**, **b**.

**Extended Data Fig. 8:**
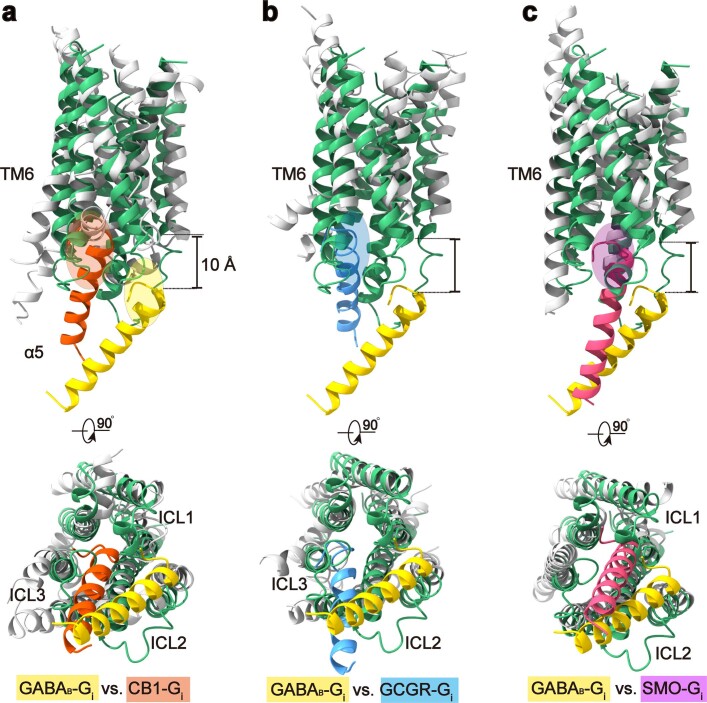
Comparison of the G_i_ binding pocket among class-A, -B, -C and -F GPCRs. **a**–**c**, Parallel comparisons of the G_i_ binding pocket between the GABA_B_ and class-A CB1 (**a**), class-B GCGR (**b**) and class-F SMO (**c**) receptors. Four structures were aligned by the class-A TMD as a reference, as in Fig. 2. A comparison of the indicated two receptors is shown. Colours for α5 are: GABA_B_-bound, yellow; CB1-bound (PDB 6N4B), orange–red; GCGR-bound (PDB 6LML), sky blue; and SMO-bound (PDB 6OT0), magenta.

G_il_ binding to GABA_B_ forms a smaller interface (856 Å^2^) than in the class-A cannabinoid receptor 1 (1,155 Å^2^), class-B glucagon receptor (905 Å^2^), or class-F smoothened receptor (1,060 Å^2^) (Extended Data Fig. 8). The α_5_ helices coupled to class-A, -B and -F GPCRs through nearly the same intracellular cavity that reached the same depth into the TMDs of the receptor, whereas the α_5_ of G_i_ coupled to GABA_B_ inserts around 10 Å less deeply (Fig. 4a, Extended Data Fig. 8). Consequently, the C-terminal end of the α_5_ of Gα_i_ did not penetrate into a central cavity (which we term pocket^R^), but rather into a cavity located at the periphery (Fig. 4b, c). The extended ICL2 inserted into a G-protein pocket (which we term pocket^G^) that comprised the α_5_ helix, the linker region in β_2_–β_3_ and the linker region of α_N_–β_1_ (Fig. 4c). The GABA_B_-bound G_i1_ adopted open conformations, showing a notable separation of Ras and helical domains and the displacement of the α_5_ helix away from GDP-binding sites (Fig. 4d). Compared with other G_il_ structures in the GPCR–G_i_ complexes, GABA_B_-activated G protein retained all of the expected conformational features of an activated G protein, except for a 25° upward rotation along the α_N_ domain that was due to the distinct interactions with ICL2 of GABA_B_ (Fig. 4e). Collectively, these results suggest that the GABA_B_ adopts a distinct mode of G-protein coupling compared to class-A, -B and -F GPCRs.

## Discussion

**Extended Data Fig. 9:**
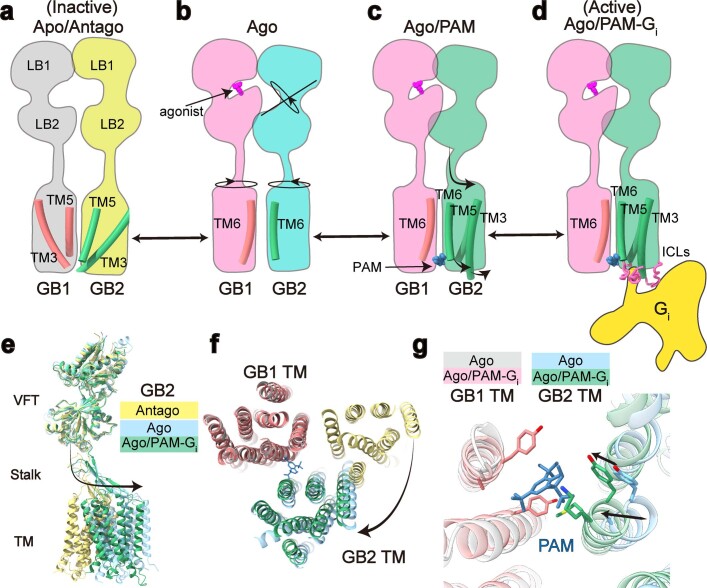
Proposed model of GABA_B_ activation. **a**–**d**, Schematic of the essential steps for GABA_B_ activation. **e**, Comparison of the relative bending of GB2 subunit in the agonist- and PAM–G_i_-bound (ago/PAM-G_i_) (green), the agonist-bound (ago) (PDB 6UO9) (blue), and antagonist-bound (antago) (PDB 7C7S) (yellow) when aligned on the GB2 VFT. **f**, The transmembrane domain rearrangement of GABA_B_ during activation. The antagonist-bound (antago) (PDB 7C7S) (yellow), agonist-bound (ago) (PDB 6UO9) (blue), and the agonist- and PAM–G_i_-bound (ago/PAM–G_i_) (green) structures of the TMD of GABA_B_ were aligned by the TMB of GB1. **g**, PAM binding in agonist- and PAM–G_i_-bound GABA_B_ (ago/PAM–G_i_).

Our cryo-EM structure of the GABA_B_–G_i_ complex stabilized with an agonist and a PAM reveals an asymmetric activation process in which a single G protein interacts with GB2. It also reveals a distinct mode of G-protein activation, as the Gα C-terminal end interacts with a shallow groove that involves TM3 and the ICLs of GB2 (Extended Data Fig. 9) rather than with a central pocket that results from TM6 movement (as observed with other GPCRs)^5,7^. Consistent with the PAM-binding site being located at the TM6 interface between the subunits, no outward movement of TM6 is observed in the GABA_B_–Gi complex. Despite this different mode of activation, the activated G protein retained all of the expected conformational changes (as observed with the other classes of GPCR)^5,27,28^. This binding mode explains the G_i_ selectivity of the GABA_B_, and is supported by numerous mutations within the ICLs and the G protein^22,25,29^. The similar determinants that are involved in G-protein recognition, the conservation of ICL2^30,31^ and the similar mode of activation of these dimeric receptors suggest that there may be a similar coupling mechanism in the other class-C GPCRs.

The agonist- and PAM–G_i_-bound GABA_B_ structure was almost identical to that observed with the agonist and PAM without G_i_ (Extended Data Fig. 5b), which indicates that the G protein has no additional effect on the conformation of the receptor. This also suggests that the PAM has an effect similar to that of the G protein on the conformation of GB2. Although an agonist-bound GABA_B_–Gi complex will be informative in clarifying this issue, we have not been able to obtain such a complex that is stable enough for cryo-EM analysis.

Our observations demonstrate how agonist binding in the VFT domain of GB1 can allosterically control activation of the TMD of GB2. Our results show that a closed VFT domain of GB1 leads to a new positioning of the VFT domain of GB2 that is associated with the bending of this subunit and the movement of the TMDs one relative to the other, which leads to a change from TM5 to TM6 as the dimer interface (Extended Data Fig. 9a, b). This probably corresponds to the first activation step, as previously reported in mGlus^32,33^. The addition of a PAM (with or without the G protein) leads to a second movement of the two TMDs with a closer apposition of GB2 on GB1 that creates the PAM-binding site and leads to a change in the bending of the GB2 subunit (Extended Data Fig. 9e–g). This bending is associated with a slight change in the conformation of ICL2 and a movement of TM3 relative to TM5 in GB2 only, which opens the shallow cavity in which the C-terminal end of the G protein binds (Fig. 2b). This model highlights the intra-GB2-subunit conformational changes that result from the closing of the VFT domain of GB1 and the contact between the TM6s as being the essential route for the allosteric interaction between the agonist-binding site and the G-protein-activating site.

Taken together, our observations provide structural information for the asymmetric activation of a dimeric GABA_B_, which may also apply to other class-C receptors. Our results also reveal that—despite a different binding mode compared to other GPCRs—activated GABA_B_ leads to an almost identical conformational change in the Gα protein that allows the receptor to act as a guanine nucleotide-exchange factor.

## Methods

No statistical methods were used to predetermine sample size. The experiments were not randomized, and investigators were not blinded to allocation during experiments and outcome assessment.

### Constructs

To facilitate expression and purification, human GABA_B_ with the haemagglutinin (HA) signal peptide—including GB1a (UniProt: Q9UBS5) and GB2 (UniProt: O75899)—were cloned into the pEG BacMam vector^34^. An 8× histidine tag and 3C protease cleavage site were inserted at the C terminus of the GB1a (residues 15–919) subunit, and a Flag epitope tag (DYKDDDD) and a 2× GSG linker were added to the N terminus of the GB2 (residues 42–819) subunit. GABA_B_ and G_il_ mutants were generated using site-directed mutagenesis. All the constructs were confirmed by sequencing.

### Expression and purification of scFv16

scFv16 was expressed and purified as previously described^35^. In brief, the 6×histidine-tagged scFv16 was expressed in secreted form in *Trichoplusia ni* Hi5 insect cells for 48 h using the Bac-to-Bac system. The expressed scFv16 was purified using a Ni-NTA resin. The C-terminal 6×His tag of the Ni-NTA eluent was cleaved by 3C protease and further purified by gel filtration chromatography using a Superdex 200 column. Finally, the purified scFv16 was concentrated and stored at −80 °C until further use.

### Expression and purification of heterotrimeric G_i1_

Heterotrimeric G_i1_ was expressed and purified as previously described^35^. In brief, the dominant-negative Gα_i1_ (S47N, G203A, E245A and A326S) and human β1γ2 subunits (β1–8×His tag) were co-expressed in Hi5 insect cells for 48 h using the Bac-to-Bac system. The cells were collected and lysed with a buffer containing 10 mM HEPES (pH 7.5), 100 μM MgCl_2_ and 10 μM GDP. The cell membrane was collected by centrifugation and heterotrimeric G_i1_ was extracted in a buffer containing 1% sodium cholate. The supernatant was purified by Ni-NTA column and the detergent was exchanged with *n*-dodecyl-β-d-maltoside (Anatrace) on a column. Afterward, G_i1_ was mixed with a 1.2 molar excess of scFv16 and further purified by Superdex 200 column. Finally, the G_i1_–scFv16 complex was concentrated and flash-frozen in liquid nitrogen until further use.

### Formation of the GABA_B_–G_i1_–scFv16 complex

The GB1 and GB2 plasmids mixed with PEI 25 K at a 3:0.5:0.5 ratio of PEI to GB1 and GB2 plasmid (w/w) were added to HEK293F cells when the density reached about 2.8 million per ml. Seventeen hours after infection, sodium butyrate was added at a final concentration of 10 mM and the cells were grown for another 3 days at 30 °C before being collected^11^. The collected cells were solubilized for 3 h at 4 °C in a buffer containing 0.5% (w/v) lauryl maltose neopentyl glycol (Anatrace) and 0.1% (w/v) cholesteryl hemisuccinate (Anatrace). After centrifugation at 30,000*g* for 30 min, the GABA_B_ was purified by Ni-NTA column and M1 anti-Flag affinity resin. The GABA_B_ was further concentrated and mixed with a 1.3 molar excess of G_i1_–scFv16 complex in the presence of 100 μM baclofen and 50 μM BHFF. The sample was incubated at 25 °C for 1 h, followed by the addition of 0.2 U ml^−1^ apyrase for an additional 1.5-h incubation at 24 °C^36^. Finally, the sample was purified using a Superose 6 Increase column (GE Healthcare) to acquire a homogeneous GABA_B_ receptor–G_i1_ complex. The entire purification procedure was accomplished in 12 h, followed by immediate verification to acquire a stable and fresh sample for structural determination.

### Cryo-EM grid preparation and data collection

To prepare cryo-EM grids, 3.0 μl of the purified baclofen- and BHFF-activated GABA_B_–G_i1_ complex at 1.8 mg ml^−1^ was applied onto the glow-discharged holey carbon grids (Quantifoil, R1.2/1.3, 300 mesh). The grids were blotted for 3.0 s with a blot force of 3 at 4 °C, 100% humidity, and then plunge-frozen in liquid ethane using Vitrobot Mark IV (Thermo Fischer Scientific). Cryo-EM data collection was performed on a Titan Krios at 300 kV accelerating voltage in the Center of Cryo-Electron Microscopy (Zhejiang University). Micrographs were recorded using a Gatan K2 Summit Detector in counting mode with a pixel size of 1.014 Å using SerialEM software^37^. Image stacks were obtained at a dose rate of about 8.0 electrons per Å^2^ per second with a defocus ranging from −1.0 to −2.5 μm. The total exposure time was 8 s, and 40 frames were recorded per micrograph. A total of 13,843 movies were collected for the GABA_B_–G_i1_ complex.

### Cryo-EM data processing

Image stacks for the GABA_B_–G_i1_ complex were subjected to beam-induced motion correction using MotionCor2^38^. Contrast transfer function parameters for non-dose-weighted micrographs were determined by Gctf^39^. Cryo-EM data processing was performed using Relion 3.1^40^ and CryoSPARC 2.15^41^. Template-based particle selection yielded 5,889,932 particle projections using Relion. The projections were imported to CryoSPARC for 2D classification to discard poorly defined particles. The selected particle projections were further subjected to ab initio reconstruction and heterogeneous refinement in CryoSPARC. The well-defined subsets accounting for 1,366,533 particles were re-extracted for further processing in Relion. Three-dimensional classification showed that G_i1_ predominantly bound to GB2, however, a small subset (112,338 particles) was also found to interact with GB1. To sort out conformational uniform particles for 3D reconstruction, these projections were subjected to 3D classification with a mask on the TMD–G_il_, producing one good subset that accounted for 362,826 particles. Further 3D classifications focusing the alignment on the G_il_ produced two good subsets, which accounted for 275,089 particles that were subsequently subjected to 3D refinement, contrast transfer function refinement and Bayesian polishing. The overall refinement of GABA_B_–G_i1_ generated a map with an indicated global resolution of 3.5 Å at a Fourier shell correlation of 0.143. To further improve the map quality of the complex (especially for G_i1_), local 3D reconstruction focusing on the GABA_B_ receptor and G_i1_ was performed using the partial signal subtracted particles in Relion. The local refinement maps for the GABA_B_ and G_i1_ showed a global resolution of 3.3 Å and 3.4 Å, respectively, which were combined on the basis of the global refinement map using ‘vop maximum’ command in UCSF Chimera^42^. This composite map of the GABA_B_–G_i1_ complex was used for subsequent model building and analysis. Global and local resolution was determined using the Bsoft 2.0.7 package^43^ with half maps as input maps.

### Model building and refinement

The model of the active GABA_B_ (PDB 7C7Q)^11^ was used to generate the initial template of the GABA_B_. The atomic coordinates of G_il_ and scFv16 from the structure of the human cannabinoid receptor 2–G_i1_ complex (PDB 6PT0)^36^ were used to generate the initial template of the G_i1_–scFv16 complex. Models of GABA_B_ and G_il_–scFv16 were docked into the electron microscopy density map using UCSF Chimera^42^. Agonist and PAM coordinates and geometry restraints were generated using a phenix.elbow^44^. The docked model was subjected to flexible fitting using Rosetta^45^ and was further rebuilt in Coot^45^ and real-space-refined in Rosetta^45^ and Phenix^44^. The final refinement statistics were validated using the module ‘comprehensive validation (cryo-EM)’ in Phenix. The goodness-of-fit of the model to the map was determined using a global model-versus-map Fourier shell correlation. The refinement statistics are provided in Supplementary Information and Extended Data Table 1. Structural figures were created using UCSF Chimera^42^ and the UCSF Chimera X package^46^.

### Enzyme-linked immunosorbent assay

The cell-surface expression of the receptor subunits was detected using an enzyme-linked immunosorbent assay (ELISA). In brief, HEK293T cells were plated in each well of a 6-well plate at a concentration of 0.3 million per ml (2 ml per well). Plasmid transfection was performed with a mixture of 200 ng Gα_i1_–lgbit, 500 ng Gγ–smbit, 500 ng Gβ, 200 ng GABA_B_ wild type (HA–GB1 and Flag–GB2) or mutants using Lipofectamine 2000 (Thermo Fisher Scientific) in 200 μl of Opti-MEM (Thermo Fisher Scientific). The Flag- and HA-tagged subunits were cotransfected into HEK293T cells and plated in a 96-well plate with a white transparent bottom. HEK293T cells were fixed with 4% paraformaldehyde and blocked with 10% fetal bovine serum (FBS). Bound antibodies coupled to horseradish peroxidase were detected by luminescence using SuperSignal ELISA Femto Maximum Sensitivity substrate (ThermoFisher Scientific), and luminescence was measured using a luminescence microplate reader (Tecan).

### IP1 accumulation assay

IP1 accumulation was measured using the IP-One HTRF kit (PerkinElmer, CisBio Bioassays). Transfected HEK293 cells were seeded in a 96-well plate, and 24 h after transfection, cells were treated with baclofen diluted in stimulation buffer in a Cisbio kit for 30 min at 37 °C. Then, cryptate-labelled anti-IP1 monoclonal antibody and d2-labelled IP1 in lysis buffer were added to the wells. After 1 h of incubation at room temperature, the plates were read in PHERAstar FS with excitation at 337 nm and emission at 620 and 665 nm. The accumulation of IP1 was calculated according to a standard dose–response curve.

### NanoBiT-G-protein dissociation assay

G-protein activation was detected using a Nanobit-G protein dissociation assay^47^. The transfection system was the same as that used in the ELISA. After 1 day of transfection, cells in the 6-well plate were digested and resuspended in complete medium DMEM (5% FBS, 1% antibiotic) and plated in 96-well flat-bottomed white microplates. After 24 h, the cells were washed twice with D-PBS and incubated in 40 μl of 5 μM coelenterazine H (Promega) solution diluted with 0.01% BSA- and 5 mM HEPES (pH 7.4)-containing HBSS (assay buffer) for 2 h at room temperature. Baseline luminescence was measured using a luminescent microplate reader (Tecan). The test compound (5×, diluted in the assay buffer) was added to the cells (10 μl) and incubated for 3–5 min at room temperature before the second measurement. The ligand-induced signal ratio was normalized to the baseline luminescence, and fold-change signals over vehicle treatment were used to show the G-protein dissociation response.

### Statistical analysis

Statistical analyses were performed on at least three individual datasets and analysed using GraphPad Prism software. Bars represent differences in the calculated agonist potency (pEC_50_), maximum agonist response (*E*_max_) and basal activity for each mutant relative to the wild-type receptor. Data are mean ± s.e.m. from at least three independent experiments, performed in triplicates. ND, not determined. *P < 0.05, **P < 0.01, ***P < 0.001, ****P < 0.0001 (one-way analysis of variance (ANOVA) followed by Dunnett’s test, compared with the response of the wild type). For dose–response experiments, data were normalized and analysed using nonlinear curve fitting for the log (agonist) versus response (three parameters) curves.

### Reporting summary

Further information on research design is available in the Nature Research Reporting Summary linked to this paper.

## Online content

Any methods, additional references, Nature Research reporting summaries, source data, extended data, supplementary information, acknowledgements, peer review information; details of author contributions and competing interests; and statements of data and code availability are available at 10.1038/s41586-021-03507-1.

### Supplementary information

Supplementary Information This file contains Supplementary Fig. 1 (the uncropped gels) and Supplementary Table 1.

Reporting Summary

Video 1 The relative motion of GABA_B_ and G_i1_ represented by the first eigenvector from the principal component analysis.

Video 2 The relative motion of GABA_B_ and G_i1_ represented by the second eigenvector from the principal component analysis.

### Source data

Source Data Fig. 2

Source Data Fig. 3

Source Data Extended Data Fig. 1

Source Data Extended Data Fig. 6

Source Data Extended Data Fig. 7

## Figures and Tables

**Extended Data Table 1:**
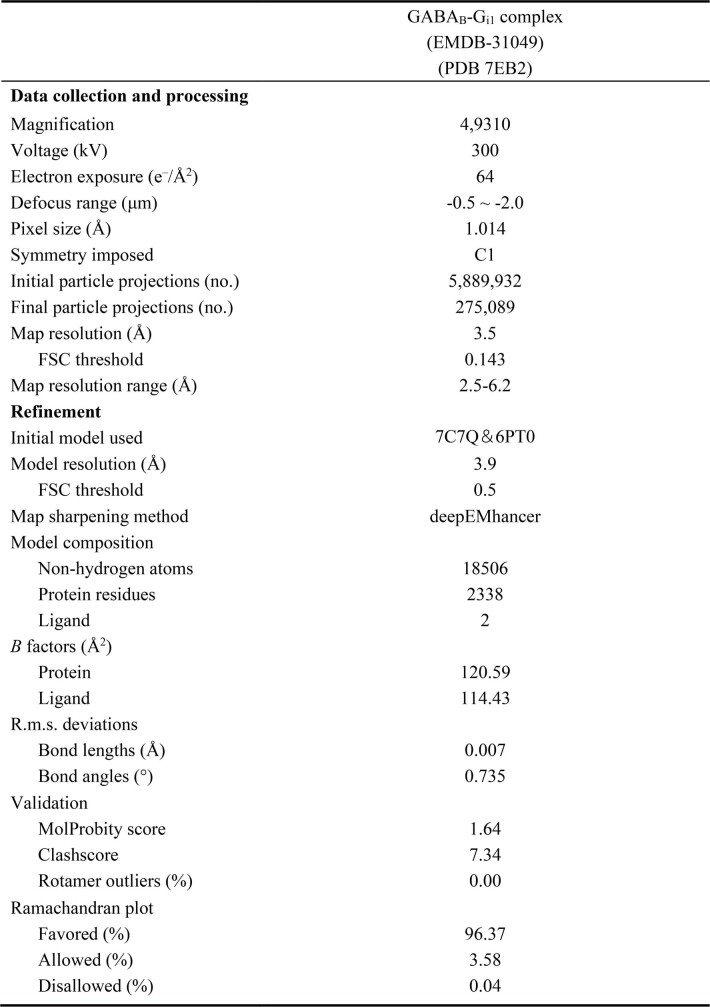
Cryo-EM data collection, model refinement and validation statistics

## Data Availability

The cryo-EM density map and corresponding atomic coordinate of the GABA_B_–G_i1_ complex have been deposited in the Electron Microscopy Data Bank and PDB under the accession codes EMD-31049 and 7EB2, respectively. All data analysed in this study are included in this Article and its Supplementary Information. Any other relevant data are available from the corresponding authors upon reasonable request. Source data are provided with this paper.

## References

[1] Hauser AS, Attwood MM, Rask-Andersen M, Schiöth HB, Gloriam DE (2017). Trends in GPCR drug discovery: new agents, targets and indications. Nat. Rev. Drug Discov..

[2] Gilman AG (1987). G proteins: transducers of receptor-generated signals. Annu. Rev. Biochem..

[3] Rasmussen SG (2011). Crystal structure of the β2 adrenergic receptor–Gs protein complex. Nature.

[4] Kang Y (2018). Cryo-EM structure of human rhodopsin bound to an inhibitory G protein. Nature.

[5] Qi X (2019). Cryo-EM structure of oxysterol-bound human Smoothened coupled to a heterotrimeric G_i_. Nature.

[6] Velazhahan V (2021). Structure of the class D GPCR Ste2 dimer coupled to two G proteins. Nature.

[7] Hilger D (2020). Structural insights into differences in G protein activation by family A and family B GPCRs. Science.

[8] Kniazeff J, Prézeau L, Rondard P, Pin JP, Goudet C (2011). Dimers and beyond: the functional puzzles of class C GPCRs. Pharmacol. Ther..

[9] Kaupmann K (1998). GABA_B_-receptor subtypes assemble into functional heteromeric complexes. Nature.

[10] Pin JP, Bettler B (2016). Organization and functions of mGlu and GABA_B_ receptor complexes. Nature.

[11] Mao C (2020). Cryo-EM structures of inactive and active GABA_B_ receptor. Cell Res..

[12] Bettler B, Kaupmann K, Mosbacher J, Gassmann M (2004). Molecular structure and physiological functions of GABA_B_ receptors. Physiol. Rev..

[13] Jones KA (1998). GABA_B_ receptors function as a heteromeric assembly of the subunits GABA_B_R1 and GABA_B_R2. Nature.

[14] White JH (1998). Heterodimerization is required for the formation of a functional GABA_B_ receptor. Nature.

[15] Chun L, Zhang WH, Liu JF (2012). Structure and ligand recognition of class C GPCRs. Acta Pharmacol. Sin..

[16] Park J (2020). Structure of human GABA_B_ receptor in an inactive state. Nature.

[17] Papasergi-Scott MM (2020). Structures of metabotropic GABA_B_ receptor. Nature.

[18] Shaye H (2020). Structural basis of the activation of a metabotropic GABA receptor. Nature.

[19] Koek W, Cheng K, Rice KC (2013). Discriminative stimulus effects of the GABA_B_ receptor-positive modulator rac–BHFF: comparison with GABA_B_ receptor agonists and drugs of abuse. J. Pharmacol. Exp. Ther..

[20] Binet V (2007). Common structural requirements for heptahelical domain function in class A and class C G protein-coupled receptors. J. Biol. Chem..

[21] Gether U (2000). Uncovering molecular mechanisms involved in activation of G protein-coupled receptors. Endocr. Rev..

[22] Havlickova M (2002). The intracellular loops of the GB2 subunit are crucial for G-protein coupling of the heteromeric γ-aminobutyrate B receptor. Mol. Pharmacol..

[23] Tu H (2010). GABA_B_ receptor activation protects neurons from apoptosis via IGF-1 receptor transactivation. J. Neurosci..

[24] Conklin BR, Farfel Z, Lustig KD, Julius D, Bourne HR (1993). Substitution of three amino acids switches receptor specificity of G_q_α to that of G_i_α. Nature.

[25] Franek M (1999). The heteromeric GABA-B receptor recognizes G-protein α subunit C-termini. Neuropharmacology.

[26] Vroling B (2011). GPCRDB: information system for G protein-coupled receptors. Nucleic Acids Res..

[27] Krishna Kumar K (2019). Structure of a signaling cannabinoid receptor 1–G protein complex. Cell.

[28] Qiao A (2020). Structural basis of G_s_ and G_i_ recognition by the human glucagon receptor. Science.

[29] Duthey B (2002). A single subunit (GB2) is required for G-protein activation by the heterodimeric GABA_B_ receptor. J. Biol. Chem..

[30] Havlickova M (2003). The second intracellular loop of metabotropic glutamate receptors recognizes C termini of G-protein α-subunits. J. Biol. Chem..

[31] Gomeza J (1996). The second intracellular loop of metabotropic glutamate receptor 1 cooperates with the other intracellular domains to control coupling to G-proteins. J. Biol. Chem..

[32] Hlavackova V (2012). Sequential inter- and intrasubunit rearrangements during activation of dimeric metabotropic glutamate receptor 1. Sci. Signal..

[33] Grushevskyi EO (2019). Stepwise activation of a class C GPCR begins with millisecond dimer rearrangement. Proc. Natl Acad. Sci. USA.

[34] Goehring A (2014). Screening and large-scale expression of membrane proteins in mammalian cells for structural studies. Nat. Protocols.

[35] Koehl A (2018). Structure of the µ-opioid receptor–G_i_ protein complex. Nature.

[36] Xing C (2020). Cryo-EM structure of the human cannabinoid receptor CB2–G_i_ signaling complex. Cell.

[37] Schorb M, Haberbosch I, Hagen WJH, Schwab Y, Mastronarde DN (2019). Software tools for automated transmission electron microscopy. Nat. Methods.

[38] Zheng SQ (2017). MotionCor2: anisotropic correction of beam-induced motion for improved cryo-electron microscopy. Nat. Methods.

[39] Zhang K (2016). Gctf: real-time CTF determination and correction. J. Struct. Biol..

[40] Scheres SH (2016). Processing of structurally heterogeneous cryo-EM data in RELION. Methods Enzymol..

[41] Punjani A, Rubinstein JL, Fleet DJ, Brubaker M (2017). A. cryoSPARC: algorithms for rapid unsupervised cryo-EM structure determination. Nat. Methods.

[42] Pettersen EF (2004). UCSF Chimera—a visualization system for exploratory research and analysis. J. Comput. Chem..

[43] Heymann JB (2018). Single particle reconstruction and validation using Bsoft for the map challenge. J. Struct. Biol..

[44] Adams PD (2010). PHENIX: a comprehensive Python-based system for macromolecular structure solution. Acta Crystallogr. D.

[45] Emsley P, Cowtan K (2004). Coot: model-building tools for molecular graphics. Acta Crystallogr. D.

[46] Goddard TD (2018). UCSF ChimeraX: meeting modern challenges in visualization and analysis. Prot. Sci..

[47] Kato HE (2019). Conformational transitions of a neurotensin receptor 1–G_i1_ complex. Nature.

