# Supplementary material for: Structural basis of GABAB receptor–Gi protein coupling
Source: Nature. 2021 Apr 28;594(7864):594–8. doi: 10.1038/s41586-021-03507-1 (PMC8222003; doi:10.1038/s41586-021-03507-1)
Supplement: Supplementary file 1 — This file contains Supplementary Fig. 1 (the uncropped gels) and Supplementary Table 1. [file 41586_2021_3507_MOESM1_ESM.pdf]

nature portfolio

https://doi.org/10.1038/s41586-021-03507-1

Supplementary information

Structural basis of $GABA_B$ receptor–G_i protein coupling

In the format provided by the authors and unedited

Structural basis of $GABA_B$ receptor-G_i protein coupling

Cangsong Shen[1,2,*], Chunyou Mao[2,3,*], Chanjuan Xu[1,4,*], Nan Jin[1,2,*], Huibing Zhang[2,3], Dan-Dan Shen[2,3], Qingya Shen[2,3], Xiaomei Wang[1], Tingjun Hou[6], Philippe Rondard[5], Zhong Chen[7], Jean-philippe Pin[5,#], Yan Zhang[2,3,8,9,#], Jianfeng Liu[1,4,#]

[1]ZJU-HUST joint laboratory of Cellular Signaling, Key Laboratory of Molecular Biophysics of MOE, International Research Center for Sensory Biology and Technology of MOST, College of Life Science and Technology, Huazhong University of Science and Technology (HUST), Wuhan, 430074, China

[2]Department of Biophysics and Department of Pathology of Sir Run Run Shaw Hospital, Zhejiang University School of Medicine, Hangzhou 310058, China.

[3]Liangzhu Laboratory, Zhejiang University Medical Center, Hangzhou 311121, China

[4]Bioland Laboratory, Guangzhou Regenerative Medicine and Health Guangdong Laboratory, Guangzhou 510005, China

[5]Institut de Génomique Fonctionnelle (IGF), Université de Montpellier, CNRS, INSERM, 34094 Montpellier, France

[6]Innovation Institute for Artificial Intelligence in Medicine of Zhejiang University, College of Pharmaceutical Sciences, Zhejiang University (ZJU), Hangzhou 310058, China

[7]Key Laboratory of Neuropharmacology and Translational Medicine of Zhejiang Province, Zhejiang Chinese Medical University, Hangzhou, China

[8]Zhejiang Provincial Key Laboratory of Immunity and Inflammatory diseases, Hangzhou 310058, China

[9]MOE Frontier Science Center for Brain Research and Brain-Machine Integration, Zhejiang University School of Medicine, Hangzhou 310058, China

[*]Equal contributions

[#]To whom corresponding should be addressed: Jianfeng Liu (jfliu@mail.hust.edu.cn); Jean-Philippe Pin (jean-philippe.pin@igf.cnrs.fr); Yan Zhang (zhang_yan@zju.edu.cn)

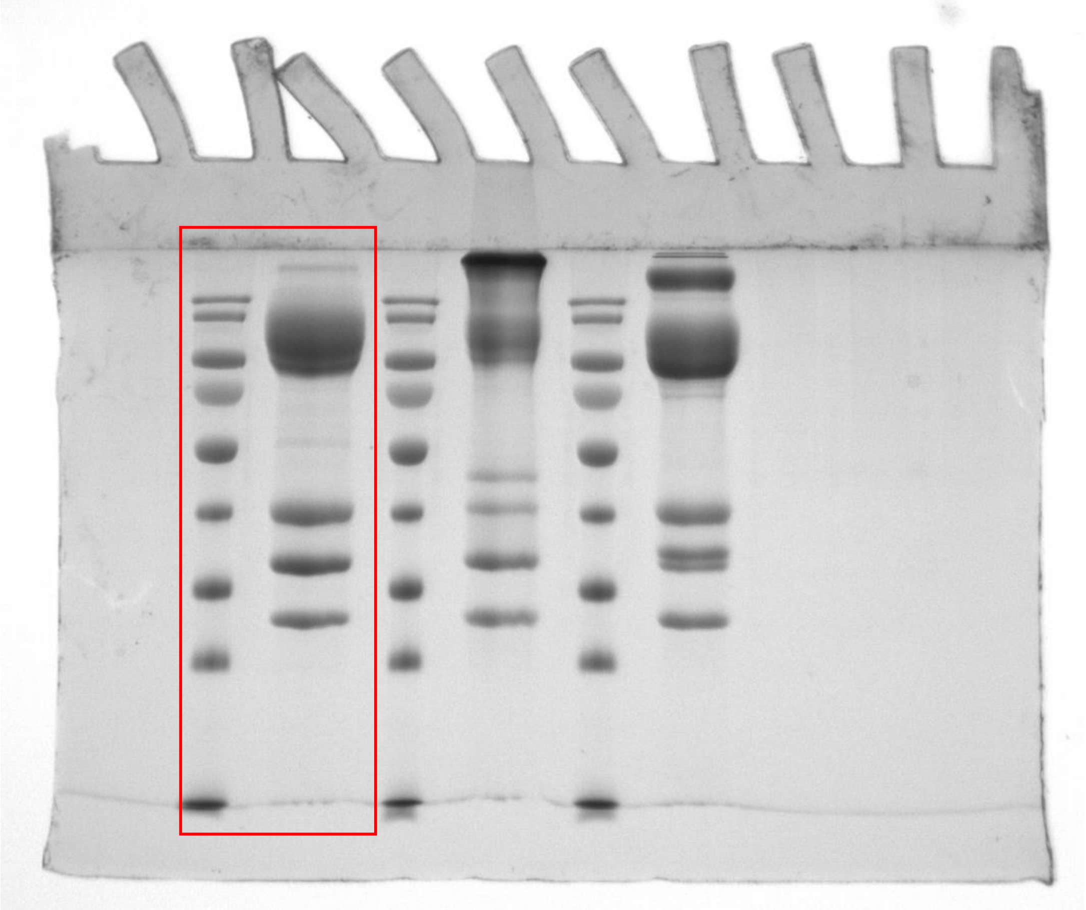

Supplementary Figure 1 | SDS-PAGE of the size-exclusion chromatography peak of GABA$_B$-G$_i$-scFv16 complex. Uncropped gel used for Extended Data Fig. 1d. The cropping box is shown in red.

			Mean ± s.e.m. (n)			
	**EC50 (μM)**	**Fold shift**	**pEC_{50}**	**E_{max}**	**Basal activity**	**Expression**
WT	0.5	1	6.3 ±0.1 (13)	100 (9)	31.4 ±3.7 (9)	100 (4)
K510A	1.0	2	6.0 ±0.2 (3)	64.0 ±6.4 (4)	15.9 ±4.5 (4)	133.8 ±7.7 (4)
K513A	1.7	4	5.8 ±0.1 (3)	71.0 ±2.1 (4)	15.5 ±3.4 (4)	119.9 ±17.9 (4)
M514A	0.3	1	6.5 ±0.2 (4)	93.2 ±7.2 (4)	27.7 ±6.2 (4)	109.3 ±5.3 (4)
S515A	nd	nd	nd	7.7 ±0.9 (4)	7.0 ±0.4 (4)	47.0 ±10.9 (4)
R577A	nd	nd	nd	8.7 ±0.9 (5)	6.5 ±1.7 (5)	141.7 ±12.8 (4)
V578A	7.2	16	5.1 ±0.0 (4)	57.1 ±5.6 (4)	20.1 ±5.1 (3)	88.1 ±10.2 (3)
A580W	0.7	2	6.1 ±0.1 (4)	75.3 ±4.9 (5)	21.8 ±6.1 (5)	139.6 ±5.5 (4)
A580R	0.7	1	6.2 ±0.1 (5)	77.4 ±4.8 (5)	32.9 ±6.9 (5)	122.5 ±4.8 (4)
I581A	13.9	31	4.9 ±0.2 (5)	27.4 ±3.7 (4)	6.4 ±2.5 (4)	146.3 ±17.5 (4)
I581W	nd	nd	nd	10.4 ±1.1 (5)	8.3 ±1.4 (6)	152.3 ±20.0 (4)
F582A	14.6	32	4.8 ±0.1 (3)	79.2 ±3.5 (3)	30.5 ±6.6 (3)	82.0 ±5.6 (3)
N584A	3.9	9	5.4 ±0.0 (4)	48.9 ±4.5 (5)	10.3 ±1.7 (5)	145.3 ±9.9 (4)
V585A	0.5	1	6.3 ±0.1 (5)	66.3 ±8.3 (6)	28.9 ±4.7 (6)	76.6 ±6.9 (4)
K586A	1.0	2	6.0 ±0.0 (3)	51.7 ±14.6 (3)	13.8 ±2.5 (3)	101.0 ±24.0 (4)
M587A	2.6	6	5.6 ±0.1 (6)	47.2 ±4.8 (6)	11.8 ±3.1 (6)	127.4 ±17.3 (4)
K588A	0.4	1	6.4 ±0.1 (3)	78.8 ±5.6 (3)	16.4 ±4.9 (3)	147.7 ±0.2 (3)
K589A	0.9	2	6.0 ±0.0 (5)	78.4 ±7.6 (4)	16.7 ±2.7 (4)	88.1 ±8.9 (4)
K590A	3.5	8	5.5 ±0.1 (6)	55.3 ±5.1 (5)	12.1 ±4.1 (5)	119.2 ±21.2 (4)
I591A	0.7	1	6.2 ±0.1 (3)	38.8 ±7.5 (3)	6.9 ±1.9 (4)	69.2 ±12.9 (3)
I592A	10.2	22	5.0 ±0.1 (4)	25.8 ±4.0 (6)	6.4 ±1.7 (6)	117.6 ±16.1 (4)
I592W	1.7	4	5.8 ±0.1 (3)	58.7 ±3.3 (4)	10.7 ±0.7 (4)	98.3 ±23.2 (4)
K593A	1.2	3	5.9 ±0.1 (4)	40.5 ±6.1 (4)	8.5 ±1.6 (3)	106.4 ±13.3 (3)
D594A	2.1	5	5.7 ±0.1 (4)	47.7 ±7.3 (4)	9.5 ±1.2 (4)	119.3 ±14.8 (4)
L686A	6.1	13	5.2 ±0.1 (5)	59.5 ±2.2 (4)	8.6 ±0.7 (4)	146.2 ±20.5 (4)

Supplementary Table 1 | Agonist-induced IP1 accumulation of wild-type and mutant $GABA_B$ receptor using the chimeric Gα protein $G\alpha_{qi9}$. The fold shift was calculated by dividing the EC50 of WT $GABA_B$. The Emax and basal activity were calculated by percentage of the maxium of baclofen-induced IP production in WT $GABA_B$. The expression level was normalized with WT $GABA_B$ surface expression detected by anti-HA antibody using Elisa assay. Data are shown as mean ± SEM from at least three independent experiments performed in technical triplicate. The numbers of independent experiments (n) are shown in the parentheses. nd (not determined) refers to data where a robust concentration response curve could not be established within the concentration range tested.
