# Supplementary material for: Structural basis of GABAB receptor–Gi protein coupling
Source: Nature. 2021 Apr 28;594(7864):594–8. doi: 10.1038/s41586-021-03507-1 (PMC8222003; doi:10.1038/s41586-021-03507-1)
Supplement: Supplementary file 2 — Reporting Summary [file 41586_2021_3507_MOESM2_ESM.pdf]

nature research

Corresponding author(s): Janfeng Liu, Jean-Philippe Pin, Yan Zhang

Last updated by author(s): Mar 21, 2021

Reporting Summary

Nature Research wishes to improve the reproducibility of the work that we publish. This form provides structure for consistency and transparency in reporting. For further information on Nature Research policies, see Authors & Referees and the Editorial Policy Checklist.

Statistics

For all statistical analyses, confirm that the following items are present in the figure legend, table legend, main text, or Methods section.

n/a	Confirmed	
☐	☒	The exact sample size (n) for each experimental group/condition, given as a discrete number and unit of measurement
☐	☒	A statement on whether measurements were taken from distinct samples or whether the same sample was measured repeatedly
☒	☐	The statistical test(s) used AND whether they are one- or two-sided<br>*Only common tests should be described solely by name; describe more complex techniques in the Methods section.*
☒	☐	A description of all covariates tested
☒	☐	A description of any assumptions or corrections, such as tests of normality and adjustment for multiple comparisons
☐	☒	A full description of the statistical parameters including central tendency (e.g. means) or other basic estimates (e.g. regression coefficient) AND variation (e.g. standard deviation) or associated estimates of uncertainty (e.g. confidence intervals)
☐	☒	For null hypothesis testing, the test statistic (e.g. F, t, r) with confidence intervals, effect sizes, degrees of freedom and P value noted<br>*Give P values as exact values whenever suitable.*
☒	☐	For Bayesian analysis, information on the choice of priors and Markov chain Monte Carlo settings
☒	☐	For hierarchical and complex designs, identification of the appropriate level for tests and full reporting of outcomes
☒	☐	Estimates of effect sizes (e.g. Cohen's d, Pearson's r), indicating how they were calculated

Our web collection on statistics for biologists contains articles on many of the points above.

Software and code

Policy information about availability of computer code

Data collection	Cryo-EM data collection was performed using SerialEM3.8.
Data analysis	The following software was used in this study: MotionCor2, Gctf1.18, RELION 3.1, CryoSPARC 2.15, Bsoft 2.0.7, Rosetta 2019.35, DeepEMhancer 1.0, Coot 0.8.9, Phenix 1.18, UCSF Chimera 1.13, UCSF ChimeraX 0.92, Graphpad Prism 7.

For manuscripts utilizing custom algorithms or software that are central to the research but not yet described in published literature, software must be made available to editors/reviewers. We strongly encourage code deposition in a community repository (e.g. GitHub). See the Nature Research guidelines for submitting code & software for further information.

Data

Policy information about availability of data

All manuscripts must include a data availability statement. This statement should provide the following information, where applicable:

- Accession codes, unique identifiers, or web links for publicly available datasets
- A list of figures that have associated raw data
- A description of any restrictions on data availability

The cryo-EM density map for the GABAB-Gi complex has been deposited in the Electron Microscopy Data Bank (EMDB) under accession codes EMD-31049 The coordinates for the model of GABAB-Gi has been deposited in the PDB under accession numbers 7EB2.
Source data have been provided including Fig.2d, 2f, 3d, 3f, 3g, Extended Fig. 1a, 6d, 6e, 7b-e, 7i and 7j.

October 2018

Field-specific reporting

Please select the one below that is the best fit for your research. If you are not sure, read the appropriate sections before making your selection.

☒ Life sciences ☐ Behavioural & social sciences ☐ Ecological, evolutionary & environmental sciences

For a reference copy of the document with all sections, see nature.com/documents/nr-reporting-summary-flat.pdf

Life sciences study design

All studies must disclose on these points even when the disclosure is negative.

Sample size	For structural determination, 13840 movies of GABAB-Gi complex were obtained using Titan Krios equipped with a Gatan K2 Summit direct electron detector. For IP1 accumulation, Elisa and Nanobit assay, at least three biologically independent experiments ($n \geq 3$) were performed in technical triplicate as indicated in related Figure legends. Data were analysed by fitting various ligand concentrations and readouts using appropriate equations in GraphPad Prism 7.0.
Data exclusions	No data was systematically excluded. The procedure of generating 3D maps from cryo-EM particles involves sorting of particles that are damaged or are false-picked that are unlikely to refine correctly. This is implemented in RELION 3.0beta.
Replication	Replication Each experiment was reproduced at least three times on separate occasions. Experimental findings were reliably reproduced.
Randomization	No randomization was attempted or needed. Randomization was not necessary as the independent variables to be tested were sufficient for the functional interpretation within this study. i.e. WT vs mutant vs control conditions or dose-response determination.
Blinding	Blinding is not necessary or valid for the purposes of structural determination. For cyro-EM study, purified GABAB-Gi complex samples were applied onto a glow-discharged holey carbon grid and subsequently vitrified using a Vitrobot Mark IV. Cryo-EM imaging was performed on a Titan Krios equipped with a Gatan K2 Summit direct electron detector. The microscope was operated at 300 kV accelerating voltage, at a nominal magnification of 29,000× in counting mode, corresponding to a pixel size of 1.014 Å.

Reporting for specific materials, systems and methods

We require information from authors about some types of materials, experimental systems and methods used in many studies. Here, indicate whether each material, system or method listed is relevant to your study. If you are not sure if a list item applies to your research, read the appropriate section before selecting a response.

Materials & experimental systems

n/a	Involved in the study
☐	☒ Antibodies
☐	☒ Eukaryotic cell lines
☒	☐ Palaeontology
☒	☐ Animals and other organisms
☒	☐ Human research participants
☒	☐ Clinical data

Methods

n/a	Involved in the study
☒	☐ ChIP-seq
☒	☐ Flow cytometry
☒	☐ MRI-based neuroimaging

Antibodies

Antibodies used	The following antibodies were used in this study: Anti-HA-Peroxidase, High Affinity (3F10)(Roche, Cat. No. 12 013 819 001, Clone BMG-3F10, rat IgG1), Monoclonal ANTI-FLAG M2-Peroxidase (HRP) (Sigma-Aldrich, Catalog Number A8592, mouse IgG1)<br><br>The Anti-HA-Peroxidase was used in 1:1000 dilution.<br>The ANTI-FLAG M2-Peroxidase was used in 1:20000 dilution.
Validation	All antibodies are well characterized and were applied according to data sheet information details.<br>Anti-HA-Peroxidase: https://www.sigmaaldrich.com/catalog/product/roche/12013819001?lang=zh&region=CN<br>ANTI-FLAG M2: https://www.sigmaaldrich.com/catalog/product/sigma/a8592?lang=zh&region=CN

Eukaryotic cell lines

Policy information about cell lines

Cell line source(s)	HEK293 cells were obtained from Cell Resource Center of Shanghai Institute for Biological Sciences (Chinese Academy of Sciences, Shanghai, China). Hi5 cells were purchased from Expression Systems (Cat 94-001S).

Authentication	All of the cell lines are maintained by the supplier. No additional authentication was performed by the authors of this study.
Mycoplasma contamination	Cell lines are tested by manufacturer for contamination.
Commonly misidentified lines<br>(See ICLAC register)	None of the cell lines used is listed in the database of commonly misidentified cell lines maintained by ICLAC.
